# Assessment of Burden and Stress Among Caregivers of Terminally Ill Patients in a Saudi University Hospital: A Cross-Sectional Study

**DOI:** 10.7759/cureus.14215

**Published:** 2021-03-31

**Authors:** Eisa Y Ghazwani, Abdullah A Al-Shehri, Faizah A Alghamdi

**Affiliations:** 1 Family and Community Medicine Department, College of Medicine, Najran University, Najran, SAU; 2 Oncology Center, East Jeddah Hospital, Jeddah, SAU; 3 Oncology Center, King Abdullah University Hospital, Jeddah, SAU

**Keywords:** palliative, saudi arabia, caregivers, burden, stress

## Abstract

Background

As the burden of cancer in Saudi Arabia has increased, the number of terminally ill patients is growing. In parallel, family caregivers’ burden has emerged as an escalating problem within the field of palliative medicine. In this study, we aimed to explore the prevalence and types of burden experienced by caregivers of terminally ill patients and the associated risk factors.

Methodology

A cross-sectional study was conducted from March 2019 to February 2020 at Najran University Hospital in southern Saudi Arabia. The study included all caregivers of terminal Saudi patients receiving palliative care. A three-part study questionnaire was used for data collection: socio-demographic characteristics, the Arabic version of Zarit Burden Interview to quantify the caregivers’ burden, and the Caregiver Distress Scale to identify and rank the different types of burden among caregivers

Results

The study included 78 caregivers of terminally ill cancer patients. Their ages ranged between 19 and 70 years, with an arithmetic mean of 39.5 years and a standard deviation of 12.9 years. The caregiver burden was reported among the majority of the participants (96.2%); the burden was mild among 46.2%, moderate among 38.5%, and severe among 11.5% of the participants. The age of caregivers who expressed moderate-to-severe burden was significantly higher than those who expressed little-to-moderate burden (44.5 ± 13.7 versus 34.5 ± 9.8 years, respectively; p < 0.001). Moderate-to-severe burden was more observed among mothers/brothers [12 (80%)] than sons [21 (53.8%)] and daughters [six (25.0%)] (p = 0.003). Regarding caregiver distress, caregivers with shorter caregiving (≤three months) and mother/brother relation to the patient had significantly higher relationship distress scores (p < 0.001). In addition, relation to the patient was significantly associated with emotional burden score (p <0.001), social impact score (p < 0.007), and personal cost score (p < 0.001).

Conclusion

Caregiving to terminally ill cancer patients is a considerably hidden problem leading to caregiver’s burden and stress.

## Introduction

In Saudi Arabia, the cancer burden is expected to grow by five to ten times by 2030 [1]. This increase requires good palliative care to cover the increase in the cancer burden. Palliative care not only targets patients but also includes improving the quality of life for caregivers of terminally ill patients [2]. The presence of terminally ill patients in the families makes them vulnerable to many physical, social, and psychological influences [3-5]. Therefore, it is crucial to study the psychological state of the caregivers of terminally ill patients.

Some international studies have shown a different psychological impact on caregivers of patients with a terminal illness. In South Korea, Lee and Cha [3] found a significant positive correlation between psychological problems and care burden. Yoo et al. [5] conducted a systematic search of 35 studies to summarize and analyze families’ experiences of end-of-life care. They found that when a patient needs high medical care/nursing, the quality of family life is low. It raised stress among caregivers. Factors significantly associated with increased caregiver burden included younger caregivers, solid tumors, and assistance with patient’s activities of daily living [6,7]. Chua et al. [8] studied the impact of this high burden and found that a high caregiver burden was associated with higher depression scores.

A recent cross-sectional study was conducted at a university hospital in Riyadh, Saudi Arabia, to assess palliative care quality in several aspects. The evaluation included the quality of psychological elements from the perspectives of healthcare professionals. Participants rated their satisfaction with palliative care quality in the psychological aspect less than their evaluation of other studied elements. The study concluded that the psychological aspects of palliative care need further research [9]. The literature review did not find any study examining the burden and psychological impact on caregivers of patients with terminal illnesses in Saudi Arabia.

This study explores the prevalence and types of burden experienced by caregivers of terminally ill patients and the associated risk factors at Najran University Hospital in southern Saudi Arabia from 2019 to 2020.

## Materials and methods

This cross-sectional study was conducted at Najran University Hospital in southern Saudi Arabia. Caregivers of terminally ill patients receiving palliative care at Najran University Hospital constituted the study population. All caregivers of terminally ill patients admitted to the hospital from March 2019 to February 2020 were included in the study. The inclusion criteria comprised all caregivers of terminally ill Saudi patients for at least one month. The researcher developed the study questionnaire. The three-part questionnaire included the following:

(a) Socio-demographic characteristics: Age, gender, educational level, employment status, patient’s diagnosis, relationship to the patient, and duration of caregiving.

(b) The Zarit Burden Interview (ZBI): The Arabic version of the short 22-item version of the ZBI was used to quantify the caregivers’ burden [10]. It is widely used, and data obtained from various studies demonstrate internal consistency, with Cronbach’s alpha values above 0.80 [11]. Its Arabic version has been translated by Abdulmaksoud [12]. Responses were graded and scored according to a five-point Likert scale: never (0), rarely (1), sometimes (2), quite frequently (3), and nearly always (4). Therefore, the maximum total score was 88 (i.e., maximum burden) and the minimum total score was 0 (i.e., no burden) [13]. The total score was interpreted as follows [10]: 0-20, little or no burden; 21-40, mild-to-moderate burden; 41-60, moderate-to-severe burden; and 61-88, severe burden.

c) Caregiver Distress Scale (CDS): This scale was developed by Cousins et al. [14] to assess caregivers’ distress. CDS is based on four validated scales: Burden, Impact of Caregiving Scale, Caregiving Burden Scale, and Frustration Scale. All four measures were internally consistent with Cronbach’s alpha values over 0.70. The scale includes 17 items divided into five sub-constructs: relationship distress (items 5, 8, 11, 13), emotional burden (items 4, 9, 12, 15), social impact (items 1, 2, 3), care-receiver demands (items 6, 14, 16), and personal cost (items 7, 10, 17). According to a five-point Likert scale, the responses were graded as: strongly disagree, disagree, neutral, agree, and strongly agree. The score of relationship sub-constructs ranged between 0 and 16 points, emotional burden score ranged between 0 and 16 points, social impact score ranged between 0 and 12 points, care-receiver demands score ranged between 0 and 12 points, and personal cost score ranged between 0 and 12 points. The researchers personally interviewed all caregivers fulfilling the inclusion criteria during the study period. The dependent variables were the caregivers’ burden and distress scores, and the independent variables were the caregivers’ socio-demographic variables.

Data entry and analysis

Collected data were verified, and then coded and entered into a personal computer. Data analysis was carried out using Statistical Package for Social Sciences, version 25 (IBM Corp, Armonk, NY, USA). Descriptive statistics calculated frequency and percentages for categorical data and mean and standard deviation for quantitative data. Chi-square test and non-parametric test (Mann-Whitney and Kruskal-Wallis) were applied to test the significance of differences according to independent study variables. P-values equal to or less than 0.05 were considered statistically significant.

## Results

This study included 78 caregivers of terminally ill cancer patients. Their age ranged between 19 and 70 years, with a mean of 39.5 years and a standard deviation of 12.9 years. More than half (58.8%) of the participants were males, and 57.7% of the participants had a higher education degree. More than one-third (38.5%) of the participants were either housewives or not working. Regarding the relationship to the patients, half of them were the patients’ sons. The duration of caregiving was three or fewer months in approximately two-thirds (65.4) of the participants. Table 1 summarizes the remaining socio-demographic characteristics.

**Table 1:** Socio-demographic characteristics of caregivers of terminally ill patients. *Mother, father, brother

	No.	%
Gender		
Male	42	53.8
Female	36	46.2
Educational level		
≤High school	33	42.3
Higher education	45	57.7
Employment status		
No/housewife	30	38.5
Solider	12	15.4
Student	12	15.4
Employee	18	23.0
Retired	6	7.7
Relation to the patient		
Son	39	50.0
Daughter	24	30.8
Others*	15	19.2
Duration of caregiving (months)		
≤3	51	65.4
>3	27	34.6

Caregivers’ burden

**Figure 1:**
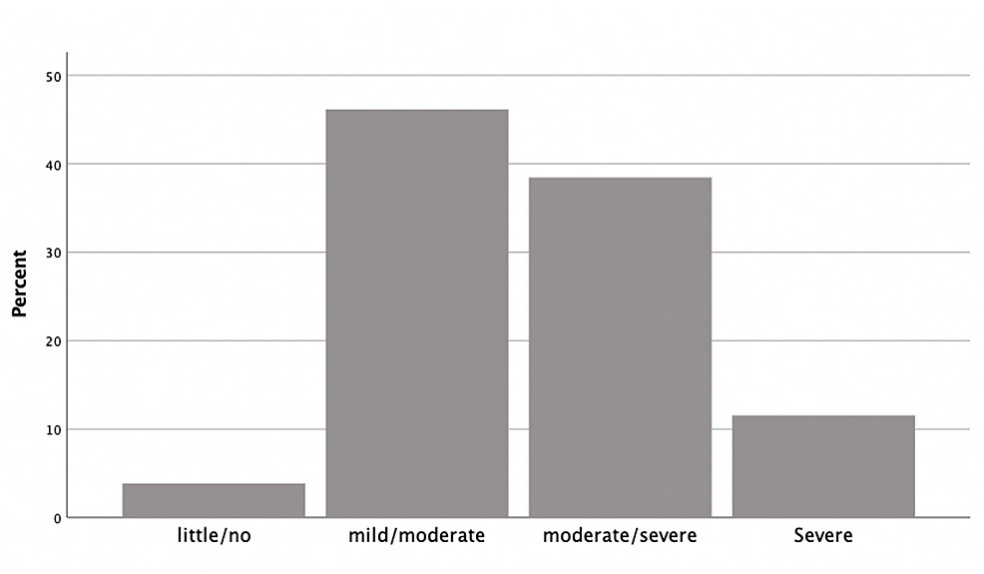
Severity of burden among caregivers of terminally ill patients.

The prevalence of burden among caregivers was reported by the majority of the participants (96.2%). The degree and frequency of the burden are shown in Figure 1.

Table 2 shows the factors associated with the caregiving burden. The age of caregivers who expressed moderate-to-severe burden was significantly higher than those who expressed little-to-moderate burden (44.5 ± 13.7 versus 34.5 ± 9.8 years, respectively; p < 0.001). Moderate-to-severe burden was reported by the majority of mothers/brothers (80%) compared to 25% of daughters (p = 0.003). Other factors were not significantly associated with caregivers’ burden.

**Table 2:** Factors associated with caregivers’ burden. SD, standard deviation *Chi-square test; **Student’s t-test; ⱡMother, brother

	Caregiver’s burden		P-Value
	Little to moderate N = 39 (%)	Moderate to severe N = 39 (%)	
Gender			
Male (n = 42)	21 (50.0)	21 (50.0)	NA*
Female (n = 36)	18 (50.0)	18 (50.0)	
Age (years)			
Mean ± SD	34.5 ± 9.8	44.5 ± 13.7	<0.001**
Educational level			
≤High school (n = 33)	15 (45.5)	18 (54.5)	0.492*
Higher education (n = 45)	24 (53.3)	21 (46.7)	
Employment status			
No/housewife (n = 30)	15 (50.0)	15 (50.0)	0.061*
Soldier (n = 12)	6 (50.0)	6 (50.0)	
Student (n = 12)	9 (75.0)	3 (25.0)	
Employee (n = 18)	9 (50.0)	9 (50.0)	
Retired (n = 6)	0 (0.0)	6 (100)	
Relation to the patient			
Son (n = 39)	18 (46.2)	21 (53.8)	0.003*
Daughter (n = 24)	18 (75.0)	6 (25.0)	
Othersⱡ (n = 15)	3 (20.0)	12 (80.0)	
Duration of caregiving (months)			
≤3 (n = 51)	24 (47.1)	27 (52.9)	0.475*
>3(n = 27)	15 (55.6)	12 (44.4)	

Caregiver Distress Scale

The results of CDS showed the median of the relationship distress score was 9 with an interquartile range (IQR) of 6-10, the median of emotional burden score was 7.5 (IQR, 6-10), the median of social impact score was 9 (IQR, 9-9), the median of care-receiver demands score was 5 (IQR, 4-7), and the median of personal cost score was 8 (IQR, 5-9).

Factors Associated With Caregiver Distress Scale

Relationship distress: The univariate analysis for factors associated with relationship distress sub-constructs revealed that the caregivers with a shorter duration of caregiving (≤three months) had higher relationship distress scores compared to those with a longer duration (>three months) (p < 0.001). The mothers/brothers of patients had higher significant scores than others (p = 0.002). Other studied factors, including gender, educational level, and employment status, were not significantly associated with the relationship distress score of the CDS (Table 3).

Emotional burden: As shown in Table 3, the relationship of the caregiver to the patients was the only significant factor for the emotional burden score of the CDS. The mothers/brothers and sons had a higher significant distress score than sons and daughters, and the distress was higher in sons than in daughters (p < 0.001). Other studied factors, including gender, educational level, employment status, and duration of caregiving, were not significantly associated with the emotional burden score of the CDS.

Social impact: As shown in Table 3, the caregiver’s employment status (p = 0.010), relation to the patient (p = 0.031), and duration of caregiving were significantly associated with the social impact scale. Other factors, including gender and educational level, were not significantly associated with social impact scores.

Personal cost: Caregiver’s gender (p = 0.012), employment status (p = 0.001) and relationship to the patient (p = 0.001) were significantly associated with the personal cost sub-construct (Table 3).

Care-receiver demands: As demonstrated in Table 3, none of the studied factors was significantly associated with care-receiver demands of the CDS.

**Table 3:** Factors associated with the sub-constructs score of the CDS. IQR, interquartile range; CDS, Caregiver Distress Scale *Mann-Whitney test; **Kruskal-Wallis test; ⱡMother, brother

	Relationship distress score		Emotional burden score		Social impact score		Care-receiver demands score		Personal cost score	
	Median (IQR)	P-Value	Median (IQR)	P-Value	Median (IQR)	P-Value	Median (IQR)	P-Value	Median (IQR)	P-Value
Gender										
Male (n = 42)	8 (7-9)	0.253*	8 (6-10)	0.238*	9 (9-9)	0.381*	5.5 (4-7)	0.495*	9 (5.75-9)	0.012*
Female (n=36)	9.5 (4-10)		6.5 (3.5-9.75)		9 (9-9.75)		5 (3.25-8.5)		7.5 (5-9)	
Educational level										
≤High school (n = 33)	9 (7-10)	0.678*	8 (6-9)	0.927*	9 (9-9)	0.917*	5 (3-7)	0.927*	8 (5-9)	0.963*
Higher education (n = 45)	9 (4-10)		7 (5-10)		9 (8-10)		5 (4-7)		8 (5-9)	
Employment status										
No/housewife (n = 30)	9.5 (4-10)	0.344**	6.5 (3-9)	0.647**	9 (9-9)	0.010**	5 (4-7)	0.107**	9 (8-9)	0.001**
Soldier (n = 12)	9 (3-9.75)		9 (3.5-10)		8.5 (6.5-9)		3.5 (0.25-7.5)		4.5 (3.25-7.25)	
Student (n = 12)	9 (3-10.5)		6.5 (5.25-10)		8.5 (6.5-11.25)		4 (3.25-7)		6 (5-10)	
Employee (n = 18)	7 (6-7)		7.5 (6-10)		9 (9-10)		5.5 (4-7)		8.5 (7-9)	
Retired (n = 6)	8 (7-9)		8.5 (8-9)		9 (9-9)		7 (7-7)		8.5 (8-9)	
Relation to the patient										
Son (n = 39)	7 (7-9)	0.002**	8 (7-10)	<0.001**	9 (9-9)	0.007**	6 (4-7)	0.732**	8 (5-9)	0.001**
Daughter (n = 24)	5.5 (3.25-10)		5.5 (1.5-6.75)		9 (8.25-9)		5 (3.25-6.75)		8 (5-9)	
Othersⱡ (n = 15)	10 (9-10)		10 (9-11)		9 (9-12)		5 (4-8)		9 (9-10)	
Duration of caregiving (months)										
≤3 (n = 51)	9 (7-10)	<0.001*	8 (6-10)	0.096*	9 (9-10)	0.031*	5 (4-7)	0.567*	8 (5-9)	0.309*
>3 (n = 27)	7 (4-9)		6 (5-8)		9 (9-9)		5 (4-7)		8 (7-9)	

## Discussion

The present work aims to determine the prevalence of burden and stress among terminally ill patients’ caregivers and identify the risk factors associated with it. The results show a high level of caregivers’ burden and distress. As terminal stages of cancer are associated with significant morbidity, caring for these patients can be burdensome for caregivers. Moreover, most of the efforts are usually directed towards patients while ignoring the caregivers’ needs and burden [8]. Therefore, understanding caregivers’ burden when providing care for terminally ill cancer patients by health professionals is essential [15] as this burden can lead to high risk of depression, reduced quality of life, work impairment, and even lead to their death [8,16,17]. Therefore, the present study was carried out as the first of its kind, to our knowledge, to explore the burden of caregivers of terminally ill patients in Saudi Arabia to target interventions at these people.

In the present study, in accordance with numerous studies [6-8,18-21], the caregiver burden was reported among the majority of the caregivers (96.2%). A severe level of burden was observed among 11.5% of the caregivers. In a study carried out in the United States [7], a high level of burden was reported among 15% of caregivers for advanced cancer patients. These results may be due to a lack of medical and societal awareness of palliative care, given its recent specialization. Consequently, the palliative service with all its components is not appropriately provided to patients and their families.

In the present study, the caregivers’ age was significantly associated with the caregiver’s burden as older caregivers experienced higher rates of moderate-to-severe burden than younger caregivers. This finding appears to contradict other studies [7,22,23] reporting that the more senior caregivers had the less severe burden of caregiving. This was due to having better mental health and a lower level of psychological distress due to caregiving. This may be explained by the nature of extended families in Saudi Arabia, where individual responsibilities increase with getting older and increase in the number of family members. With this increase in responsibilities, the natural psychological and physiological effects that occur during aging, and a person with special needs in the family, the service provider’s psychological burden may increase with increasing age. Another reason for the difference in the results may be the small sample size of the study.

In the present study, caregivers’ mean age was 39.5 ± 12.9 years, which was younger; hence, even the older caregivers were younger than those reported in previous studies [7,22,23] as the caregivers’ age ranged between 43.8 and 63.1 years. The current study also showed the burden was more among participants who had lower education, retired, and shorter caregiving duration than their counterparts with no significant association with caregiving burden. Mostly, the relatively small sample size of the present study could have a significant role in our findings. A similar study carried out in the United States [7] employed caregivers and those caring for patients requiring help and reported a higher risk of severe caregiver burden.

Regarding caregiver distress, caregivers with a shorter caregiving duration (≤three months) and caregiving by mothers/brothers had significantly higher relationship distress scores in the present study. In the current study, the caregiver’s relationship to the patients was significantly associated with the emotional burden score of the caregivers’ distress as mothers/brothers and sons had higher significant scores compared to daughters. In other studies, the burden was more among spouses than non-spousal caregivers [24,25].

In the present study, the caregiver’s gender was only associated with the personal cost of caregivers’ burden, where males had the higher burden. This coincides with the findings of another study [26], which is quite expected.

Different associated factors with caregivers’ burdens have been reported in various studies. In a systematic review by Ge and Mordiffi [6], moderate evidence supported that younger caregivers, solid tumors, and assistance with patients’ daily living activities were significantly associated with high caregiver burden. In South Korea, Yoon et al. [27] reported that longer time spent providing care per day, fewer weekly visits from other family members, low-income family functioning, and low self-esteem were modifiable factors significantly associated with caregiver burden. In addition, low monthly income and spouses being the family caregivers were non-modifiable factors. Lee et al. [28] showed that a high caregiver burden could lead to a preference for palliative care in both patients and their caregivers, potentially threatening patient autonomy. The difference in results between various studies could be attributed to the differences in tools utilized, methodology, cultural norms, and the caregivers’ socio-demographic characteristics [29]. In addition to the disease stage, some studies included only those with early cancer stages [26].

The caregiver’s burden could be related to unmet needs such as health and psychological problems, family and social support, information, religious and spiritual needs, and practical support needs [3]. Therefore, healthcare staff needs to be more aware of patients’ and their families’ experiences and potential needs [5]. In this study, we did not investigate the etiology of caregivers’ burden as we only focused on the magnitude and description of the problem and identification of some associated factors.

Although it is unique in Saudi Arabia, the present study has some important limitations that should be acknowledged. First, the small sample size was relatively small due to limited awareness about palliative care services in Saudi Arabia among patients, caregivers, and healthcare providers. Second, as the study was conducted in a single healthcare facility, there could be difficulties in generalizing the results in other healthcare facilities in the Najran region. Third, meaningful information about caregivers was missing, such as social support (formal/informal) and their health status. Despite these limitations, this study’s results shed light on this ignored and important subject by exploring the magnitude of caregiving burden among the participants and identifying some factors associated with that burden, which will help clinicians and researchers target interventions at those most in need.

## Conclusions

The burden and distress associated with caregiving to terminally ill cancer patients is a considerable and prevalent hidden problem. Older caregivers experienced higher rates of moderate-to-severe burden than younger caregivers. The significant factor associated with caregiver burden was being a mother or a brother to the ill patient. Additionally, sons were found to have a higher burden than daughters. Concerning caregiver distress, caregivers with a shorter caregiving duration (≤three months) had significantly higher relationship distress. In contrast, the caregiver’s relationship with the patients, particularly mothers and brothers, was related to the their emotional distress. Additionally, male caregivers had a significantly higher emotional distress score than female caregivers.
